# Requirement for a standardised definition of advanced gastric cancer

**DOI:** 10.3892/ol.2013.1672

**Published:** 2013-11-11

**Authors:** ANGELO DE SOL, STEFANO TRASTULLI, VERONICA GRASSI, ALESSIA CORSI, IVAN BARILLARO, ANDREA BOCCOLINI, MICOL SOLE DI PATRIZI, GIORGIO DI ROCCO, ALBERTO SANTORO, ROBERTO CIROCCHI, CARLO BOSELLI, ADRIANO REDLER, GIUSEPPE NOYA, SEONG-HO KONG

**Affiliations:** 1 Department of General Surgery, University of Perugia, St. Maria Hospital, Terni, Italy; 2 Department of General and Oncological Surgery, University of Perugia, Perugia, Italy; 3 Department of Surgical Sciences, Sapienza University of Rome, Rome, Italy; 4 Department of Surgery, Seoul National University College of Medicine, Seoul, Republic of Korea

**Keywords:** Japanese Gastroenterological Endoscopic Society, early gastric cancer, advanced gastric cancer, stomach cancer

## Abstract

Each year, ~988,000 new cases of stomach cancer are reported worldwide. Uniformity for the definition of advanced gastric cancer (AGC) is required to ensure the improved management of patients. Various classifications do actually exist for gastric cancer, but the classification determined by lesion depth is extremely important, as it has been shown to correlate with patient prognosis; for example, early gastric cancer (EGC) has a favourable prognosis when compared with AGC. In the literature, the definition of EGC is clear, however, there is heterogeneity in the definition of AGC. In the current study, all parameters of the TNM classification for AGC reported in each previous study were individually analysed. It was necessary to perform a comprehensive systematic literature search of all previous studies that have reported a definition of ACG to guarantee homogeneity in the assessment of surgical outcome. It must be understood that the term ‘advanced gastric cancer’ may implicate a number of stages of disease, and studies must highlight the exact clinical TNM stages used for evaluation of the study.

## Article

At present, stomach cancer is among the most common malignant neoplasms, with >988,000 new cases reported worldwide each year (1). However, incidence rates are markedly decreasing (2) with variations in the geographical distribution and the anatomical localisation of the disease in the various regions of the intestines (3,4). The highest incidence rates to date are in Japan and other East Asian countries, including Korea and certain regions of Japan. In addition, a number of Latin American populations are also at high risk of developing the disease, including those of Chile and Costa Rica. With regard to Europe, the highest mortality and incidence rates are observed in Hungary, Finland, Austria and Iceland (5).

Carcinoma of the stomach represents the fifth most common cause of cancer-related mortality in Western populations, despite its decreasing incidence in Europe and the United States. Gastric cancer is rarely detected (~8%) in the form of early gastric cancer (EGC) during the mildly symptomatic or asymptomatic phases. In the majority of patients, gastric cancer is diagnosed at a later stage than the first stage of disease progression (6), with classic symptoms of weight loss, consistent and dull pain in the epigastrium, loss of appetite, nausea, vomiting and chronic bleeding (7,8). Rare symptoms of disseminated gastric cancer manifest as renal colic (9). In Europe, gastric cancer represents 8–9% of newly diagnosed cancer cases, and incidence and mortality are twice as high in males compared with females. The highest incidence rates occur in Portugal, Italy, Germany and Spain (10). Various methods of pathological classifications exist for stomach cancer, including microscopic (11), macroscopic (12), biological behaviour (13,14) lesion depth [these are ECG and advanced gastric cancer (AGC)] and TNM classification (Table I) (15).

The classification determined by lesion depth is extremely important as it has been shown to correlate with patient prognosis; for example, EGC has a favourable prognosis when compared with AGC (16). EGC refers to those adenocarcinomas with growth that is limited to the mucosa or submucosa, independent of regional lymph node (LN) metastases (17). According to the Japanese Gastroenterological Endoscopy Society (18), EGC may be further classified based of the macroscopic aspect of the neoplasia into three main types, 0-I, 0-II and 0-III, of which, type 0-II has three subtypes, 0-IIa, b and c (15). The characteristics of these types are as follows: i) 0-I (protruding), polypoid tumours that protrude above the mucosa surface >3 mm; ii) 0-II (superficial), tumours with or without minimal elevation or depression relative to surrounding mucosa. Specifically, 0-IIa (superficially elevated), slightly elevated tumours that thicken the mucosa to <3 mm in thickness; 0-IIb (superficially flat), tumours without elevation or depression; and 0-IIc (superficially depressed), slightly depressed tumours; and iii) 0-III (excavated), tumours with a deep depression.

The average time between EGC diagnosis and progression is ~37 months (19) and it commonly takes 8 months for EGC to progress to an advanced stage of disease. The percentage of EGC cases reported in Japan is higher (30–50%) compared with Western countries (5–15%), where screening programmes have not been implemented (20). For example, in Korea the proportion of T1 and N0 tumours was demonstrated to increase gradually with time, from 24.8 to 41.3% in period 1 (1986–1990) and from 48.9 to 58.6% in period 4 (2000–2006; both P<0.001) (21). The importance of correctly identifying EGC is highlighted by its correlation with the excellent results achieved by surgical treatment and the favourable prognosis of EGC patients following surgery.

The survival rate following surgical resection is 70–95% depending on LN involvement. Therefore, it is extremely useful to understand the involvement of LNs prior to surgery. However, there is no simple, high sensitivity test available to evaluate LN status pre-operatively. The present diagnostic imaging techniques remain unsatisfactory, however, double contrast-enhanced ultrasonography shows promise as a new, non-invasive, convenient and reproducible method for the evaluation of LN metastasis in EGC and the prognosis of EGC pre-operatively. The identification of EGC by marked hyperenhancement allows for closer post-operative follow-up and possibly the use of drugs for the targeted growth of new vessels to prevent recurrence (22).

Macroscopic classification is of extreme prognostic relevance, therefore, it is fundamental to standardise the definitions of EGC and AGC. The definition of EGC is well-codified in the literature, however, a clear definition of AGC is required.

There are certain examples of heterogeneity in the definition of AGC in the literature, and the majority highlight an unclear or heterogeneous definition of AGC. A non-systematic search for studies that highlight a clear definition of AGC confirmed the lack of consistency for a definition of AGC in various research fields. The definitions of AGC generated in single studies are reported in Table II. The aim of the current study was to individually analyse all parameters of the TNM classification for AGC reported in each previous study.

The definitions of AGC provided in the previous singular studies were compared with the current classifications of the Japanese Society of Gastric Cancer (15) using only the parameters for TNM classification. The results showed that only 5 studies resulted in a AGC T3 or T4 classification of the gastric neoplasms (23–27), whilst the remaining studies were hypothesised to be AGC neoplasms with parietal invasion initiating from stage T2 (Table III).

With regard to parameter N, all studies, with the exception of the studies by Ott *et al*(23), Scaringi *et al*(24), Luinetti *et al*(28), Carboni *et al*(27), Bataille *et al*(26) and Theuer *et al*(29), defined gastric neoplasias as AGC even in the absence of LN involvement (N0). By contrast, in the studies by Ott *et al*(23), Scaringi *et al*(24), Carboni *et al*(27), Orditura *et al*(30) and Bataille *et al*(26), LN involvement at a minimum of the N1 stage was required for the definition of AGC. Studies by Luinetti *et al*(28) and Theuer *et al*(29), however, defined AGC neoplasms as between T2 and T4, irrespective of the N parameter (Table IV).

Macroscopic classification of EGC and AGC is a simple and reliable method to prediction LN metastasis and determine the extent of LN dissection, unless endoscopic ultrasound (EUS) or CT scans show highly suspected LN metastasis.

All studies analysed in the current study, with the exception of those by Scaringi *et al*(24), Luinetti *et al*(28), Carboni *et al*(27) and Theuer *et al*(29), defined AGC as a gastric neoplasm irrespective of the presence or absence of metastasis (M1 or M0). The exceptions do not consider parameter M at all in the definition of AGC. The studies by Orditura *et al*(30) and Ott *et al*(23) clearly reported that they considered AGCs as neoplasias, even in the absence of metastases. Finally, a study by Bataille *et al*(26) reported M1 cases as AGCs.

In the Japanese Classification of Gastric Cancer (JCGC; 3rd English edition), stomach tumours are grouped by macroscopic classification into 2 types, superficial tumours (T1) and advanced tumours (T2-4) (15).

Superficial tumours, defined as type 0 are divided into 5 subtypes according to the Japanese Endoscopy Society Classification (1962; Table V), whereas AGC is divided into 5 types according to macroscopic appearance (Table VI). According to the Japanese Classification, stages Ia, Ib, IIa and IIb (T1a, T1b and N) are associated with EGC (Table VII).

In previous Japanese studies, AGC classification has been clear and univocal. By contrast, studies of other populations often exhibit heterogeneous definitions of AGC. Non-uniformity in the definition of AGC with regard to parameter T emerged from the studies analysed previously (Table III), with 11/16 studies (68.7%) considering that AGC started from T2, whilst 5/16 studies (31.2%) considered the start from T3.

Non-homogeneity of the definition for AGC is more evident when considering the parameters N and M. The definition prescinds from LN involvement or the presence of metastases. In the studies included in the current review, 9/16 were consistent with this definition of AGC. However, 5/16 (31.2%) considered N+ tumours as AGCs and 2/16 studies (12.5%) did not evaluate LN involvement.

For parameter M, 9/16 studies (56.2%) were consistent with the definition of AGC highlighted in Western studies, and such heterogeneity of the definition was reflected again in the assessment of surgical outcome, choice of lymphadenectomy type and prognosis. It is difficult to standardise the results and outcome for survival if there is no conformity of the definition. Specific studies that do not take T2 into consideration for the classification of AGC substages. Consequently, results associated with chemotherapy and survival following surgery or the type of lymphadenectomy are likely to be false and incomparable.

The hypothesis that AGC starts from T3 leads to exclusion of T2 neoplasms from studies and is likely to modify the results. In addition, with regard to LN involvement and metastases, to consider only M+ or N+ as associated with AGC excludes N0 and M0 and leads to incomparable results.

In the context of such heterogeneity, it is impossible to use macroscopic classification since it is not certain that all studies identify neoplasms as starting from T2. Therefore, a number of studies incorrectly determine certain advanced neoplasms as early. In addition, other studies exclude neoplasms defined as advanced, therefore, making the interpretation of the results obtained difficult.

The TNM classification was standardised in 2008. The AJCC Cancer Staging manual (7^th^ edition) (31) and the JCGC (3rd English Edition) (15) are comparable, with the only differences being with regard to the M parameter. In the AJCC classification, MX is excluded and M1 includes positive peritoneal fluid cytology. However, there are no differences between the two classifications with regard to staging. When the stage is used for analysis, a c-prefix must be added to specify that the stage is a clinically predicted stage, for example, analysis by TNM stage, including cT1N0, cT2-4N+ and cM1. The definition of EGC is extremely important for prognostic purposes and for the choice of therapeutic strategy, however, the principal classification used has been the TNM classification, with univocal results. Macroscopic classification is less used and often inconsistent with the definition highlighted by the Japanese Gastric Cancer Association (JGCA).

Therefore, we recommend standardisation of the definition for AGC. To correctly evaluate the results of the previous studies, the classification supplied by the JGCA must be used to guarantee homogeneity in the assessment of surgical outcomes. It is evident that a comprehensive systematic literature search must be performed for all studies that report a definition of ACG, in order to confirm the hypothesis and to subsequently identify a uniform definition of AGC to be used worldwide for the management of patients. The cTNM stages must be identified in the systemic review for a detailed description, as EUS or CT may now be utilised to pre-operatively evaluate T and N stage, although not always with 100% accuracy; therefore, the KLASS-01 study used ‘stage 1’ cancer as an indication (32).

## Conclusion

The definition of EGC and AGC by macroscopic classification regardless of LN metastasis, is made when the tools to determine LN metastasis (EUS and high quality CT) are unavailable and gastroscopy and sonography become the most reliable methods for the evaluation of clinical stages. However, this has been an extremely useful definition for the determination of the treatment strategy and the prediction of LN metastasis and the prognosis. Although EUS and CT are capable of predicting LN metastasis (although not perfectly), the definition of EGC remains useful in a number of cases, since the majority of EGC do not exhibit LN metastasis.

Use of the term ‘advanced’ is more complicated compared with EGC, since it may be defined as: i) T2 or deeper cancer according to the Japanese classification; ii) a bulky tumour with LN enlargement compared with an ‘early stage tumour’; iii) T4b (adjacent organ invading) tumour or unresectable tumour due to adjacent organ invasion or bulky LNs without distant metastasis, often referred to as ‘locally advanced gastric cancer’; iv) M1 cancer +/− unresectable M0 cancer referred to as ‘far advanced gastric cancer’ in the majority of studies on palliative chemotherapy; or v) ‘locally advanced gastric cancer’, which may implicate ‘resectable’ cancer when compared with M1 ‘advanced’ cancer (33).

Therefore, it may be difficult to differentiate between resectable advanced stage, unresectable M0 and M1 cancers using the term ‘advanced’. It must be understood that ‘advanced gastric cancer’ may implicate a number of stages of disease and studies must highlight the exact clinical TNM stages for evaluation of the study.

## Figures and Tables

**Table I:** TNM classification.

Classification	Description
T, size and extent of the tumour	
X	Depth of tumour unknown
0	No evidence of primary tumour
1	Tumour confined to M or SM
1a	Tumour confined to M
1b	Tumour confined to SM
2	MP tumour invasion
3	SS tumour invasion
4	Tumour invasion contiguous to or exposed beyond SE/SI
4a	Contiguous to or penetrates SE and PERC exposure
4b	SI invasion
N, LNs	
X	Regional LNs cannot be assessed
0	No regional LN metastasis
1	Metastasis in 1–2 regional LNs
2	Metastasis in 3–6 regional LNs
3	Metastasis in ≥7 regional LNs
3a	Metastasis in 7–15 regional LNs
3b	Metastasis in ≥16 regional LNs.
M, distant metastasis	
X	Distant metastasis status unknown
0	No distant metastasis
1	Distant metastasis

Japanese Classification of Gastric Carcinoma [3^rd^ English edition (10)]. M, musoca; SM, submucosa; MP, muscularis propria; SS, subserosa; SE, serosa; SI, adjacent structures; PERC, peritoneal cavity.

**Table II:** AGC definition in different studies.

First author, year (ref.)	Definition
Ott *et a*l, 2003 (23)	Locally-advanced gastric cancer: cT3-4, N+ and M0
	Locally-advanced disease: cT3-4, Nx and M0
Schuhmacher *et al*, 2001 (25)	Locally-advanced gastric cancer: Stages IIIA, IIIB and IV
Chiaravalli *et al*, 2001 (34)	Advanced cancers: From T2-T4; stages IB-IV
MacDonald *et al*, 2008 (35)	Advanced gastric cancer: From T2-4; from stage IB
Piso *et al*, 2000 (36)	Advanced tumour stages III–IV; less, stages I–II and more, stages III–IV
Scaringi *et al*, 2008 (24)	Locally-advanced gastric cancer: pT3-4, N+/− and M+/−
Luinetti *et al,* 1998 (28)	EGC: Gastric cancer that is limited to the mucosa or submucosa;
	AGC: Cancer that has deeply invaded the gastric wall
Carboni *et al,* 2005 (27)	Advanced cases: T3-4 and N+
Zilberstein *et al,* 2004 (37)	Early: T1 and any N and M.
	Advanced: T2-4 and any N and M.
Doglietto *et al*, 2000 (38)	Advanced stage: Stage III
Piessen *et al*, 2009 (39)	Advanced stage: From T2; stages IIIB-IV
Soyuer *et al*, 2010 (40)	Locally-advanced: From T2; stages II–IV
Orditura, 2010 (30)	Locally-advanced gastric cancer: From T2; stage IIIA
Wirtz *et al*, 1998 (41)	Advanced carcinomas: pT2, pT3-4 and any N and M
Bataille *et al*, 2003 (26)	Locally-advanced gastric carcinoma: cT3, cT4, N+ and M+
Theuer *et al*, 2002 (29)	Advanced stage: T2-3

AGC, advanced gastric cancer; EGC, early gastric cancer.

**Table III:** Characteristics of the studies obtained for the evaluation of the AGC definition.

First author, year (ref.)	Country	n	Staging	Journal	Type of study	Definition
Ott *et al*, 2003 (23)	Germany	49	CT scans, endosonography and laparoscopic	Gastric Cancer	Clinical trial	Intoduction
Schuhmacher *et al,* 2001 (25)	Germany	42	Endoscopy, EUS, CT scan, laparoscopy	Cancer	Clinical trial	Methods
Chiaravalli *et al,* 2001 (34)	Italy	185	Histophatological, histochemical and molecular findings	Virchows Arch	Retrospective study	Results
MacDonald *et al,* 2007 (35)	Canada	64	Chest X-ray, abdominal US, CT, histology	Hum Pathol	Case-control study	Results
Piso *et al,* 2000 (36)	Germany	532	Intraoperative information, pathology report	Ann Surg Oncol	Retrospective study	Results
Scaringi *et al,* 2008 (24)	France	37	Histology	Eur J Surg Oncol	Retrospective study	Introduction
Luinetti *et al,* 1997 (28)	Italy	130	Histology	Hum Pathol	Retrospective study	Materials and methods
Carboni *et al,* 2005 (27)	Italy	20	Endoscopy with biopsy, abdominal US, CT scan	Gastric Cancer	Retrospective study	Discussion
Zilberstein *et al,* 2004 (37)	Brazil	100	Histological examination	Gastric Cancer	Retrospective study	Results
Doglietto *et al,* 2000 (38)	Italy	639	Histological examination	World J Surg	Retrospective study	Results
Piessen *et al,* 2009 (39)	France	215	Laboratory tests, esophagogastroduodenal barium study, digestive endoscopy with biopsies and CT of the thorax, mediastinum and abdomen and EUS with guided biopsies	Ann Surg Oncol	Case-control study	Results
Soyuer *et al,* 2010 (40)	Turkey	49	Histology	Pathol Res Pract	Retrospective study	Methods
Orditura *et al,* 2010 (30)	Italy	29	Histology	Arch Surg	Clinical trial	Methods
Wirtz *et al,* 1998 (41)	Germany	126	Histology	Clin Cancer Res	Retrospective study	Materials and methods-results
Bataille *et al*(26)	Germany	25	Endoscopy, endosonography, CT scan	Mol Pathol	Retrospective study	Materials and methods
Theuer *et al*(29)	USA	38	Histology	Arch Surg	Retrospective case-case (blinded) study	Results

AGC, advanced gastric cancer; EUS, endoscopic ultrasound.

**Table IV:** TNM classification for study group C.

First author, year (ref.)	T1	T2	T3	T4	N0	N1	N2	N3	M0	M1	MX
Ott *et al*, 2003 (23)			X	X		X	X	X	X		
Schuhmacher *et al,* 2001 (25)			X	X	X	X	X	X	X	X	X
Chiaravalli *et al,* 2001 (34)		X	X	X	X	X	X	X	X	X	X
MacDonald *et al,* 2008 (35)		X	X	X	X	X	X	X	X	X	X
Piso *et al*, 2000 (36)		X	X	X	X	X	X	X	X	X	
Scaringi *et al*, 2008 (24)			X	X		X	X	X			
Luinetti *et al*, 1998 (28)		X	X	X							
Carboni *et al*, 2005 (27)			X	X		X	X	X			
Zilberstein *et al,* 2004 (37)		X	X	X	X	X	X	X	X	X	
Doglietto *et al,* 2000 (38)		X	X	X	X	X	X	X	X	X	
Piessen *et al,* 2009 (39)		X	X	X	X	X	X	X	X	X	
Soyuer *et al*, 2010 (40)		X	X	X	X	X	X	X	X	X	
Orditura *et al*, 2010 (30)		X	X	X		X	X	X	X		
Wirtz *et al*, 1998 (41)		X	X	X	X	X	X	X	X	X	
Bataille *et al,* 2003 (26)			X	X		X	X	X		X	
Theuer *et al*, 2002 (29)		X	X	X							

**Table V:** Subclassification of type 0 (10).

Type (location)	Example
0-I (protruding)	Polypoid tumours
0-II (superficial)	Tumours with or without minimal elevation/depression relative to surrounding mucosa
0-IIa (elevated)	Slightly elevated tumours
0-IIb (flat)	Tumours without elevation/depression
0-IIc (depressed)	Slightly depressed tumours
0-III (excavated)	Tumours with deep depression

**Table VI:** Macroscopic types (10).

Type	Classification
0 (superficial)	Typical of T1 tumours.
1 (mass)	Polypoid tumours, sharply demarcated from the surrounding mucosa.
2 (ulcerative)	Ulcerated tumours with raised margins surrounded by a thickened gastric wall without clear margins.
3 (infiltrative ulcerative)	Ulcerated tumours with raised margins surrounded by a thickened gastric wall without clear margins.
4 (diffuse infiltrative)	Tumours without marked ulceration or raised margins, the gastric wall is thickened and indurated and the margin is unclear.
5 (unclassifiable)	Tumours that cannot be classified into any of the additional types.

**Table VII:** Stage groupings (10).

Stages	N0	N1	N2	N3
T1a (M), T1b (SM)	IA	IB	IIA	IIB
T2 (MP)	IB	IIA	IIB	IIIA
T3 (SS)	IIA	IIB	IIIA	IIIB
T4a (SE)	IIB	IIIA	IIIB	IIIC
T4b (SI)	IIIB	IIIB	IIIC	IIIC
M1 (any T and N)	IV	IV	IV	IV

M, musoca; SM, submucosa; MP, muscularis propria; SS, subserosa; SE, serosa; SI, adjacent structures.
